# Brain Health Screening in Israel’s Diverse Population

**DOI:** 10.1002/alz.091749

**Published:** 2025-01-03

**Authors:** Rafi Haddad, Rachel Ben‐Hayun, Rawan Ayoub, Yarovinsky Natalya, Essam Shihada, Merav Shor, Michal Levavi, Tali Fisher, Judith Aharon‐Peretz, David Tanne

**Affiliations:** ^1^ Global Brain Health Institute, University of California, San Francisco, San Francisco, CA USA; ^2^ Rambam Health Care Campus, Haifa Israel; ^3^ Stroke and Cognition Institute, Rambam Healthcare Campus, Haifa Israel; ^4^ Rambam Healthcare Campus, Haifa Israel; ^5^ Stroke and Cognition Institute, Rambam healthcare campus, Haifa, Israel, Haifa Israel

## Abstract

**Background:**

Israeli population is primarily comprised of Jews (74%) and Arabs (21%). Previous studies suggested a higher incidence of cerebrovascular risk factors (CVRF) and dementia among Israeli Arabs. We evaluated potential cognitive disparities between community‐dwelling Arabs and Jews diagnosed with at least one CVRF.

**Method:**

96 participants (age: 66.6± 5.3; education: 14.8± 4.1), 52 Arabs (age: 64.4± 5; education: 15.3±2.6), and 44 Jews (age: 69.1± 4.4; education: 14.8± 4.1) without known cognitive impairment and with at least one CVRF were prospectively enrolled. All participant completed a cognitive symptoms questionnaire (in their primary language) and underwent the Montreal Cognitive Assessment (MoCA) and the Tablet‐based Cognitive Assessment Tool (TabCAT) Brain Health Assessment (BHA) battery, including the TabCAT Favorites (associative memory), Match (executive functions and processing speed) and Line Orientation (visuospatial skills) tasks. Demographic and clinical characteristics were analyzed using the Student’s t‐test for continuous variables and Chi‐squared tests for categorical data.

**Results:**

Arab participants were younger (p≤0.001) than Jews, education was similar (p = 0.26). Cognitive complaints were reported by 32 (33%) participants, (24% Arab,9% Jews). Participants with cognitive complaints exhibited significantly lower scores on Match (p = 0.005), but not on Favorites (p = 0.28), Line Orientation (p = 0.48), and MoCA (p = 0.62). Arabs scored significantly worse on Match (p = 0.006) while Jews scored worse on Line Orientation (p = 0.004). Performance was similar on the Favorites (p = 0.4) and MoCA (p = 0.2). When focusing on participants with cognitive complaints Arabs exhibited significantly lower scores on Match (p = 0.036) while Jews had lower scores on Line Orientation (p = 0.06). No significant differences were observed on Favorites (p = 0.4) or MoCA (p = 0.2). Among participants without cognitive complaints no significant differences were observed between Jews and Arabs on Match (p = 0.29), Favorites (p = 0.99) or MoCA (p = 0.65). Jews had lower scores on Line Orientation (p = 0.04).

**Conclusion:**

TabCAT‐BHA proved superior to MoCA in depicting subtle cognitive impairment in people with CVRF. Despite being younger, Arabs exhibited higher prevalence of cognitive complaints and were more impaired on executive functions while Jews on visuospatial tasks.

